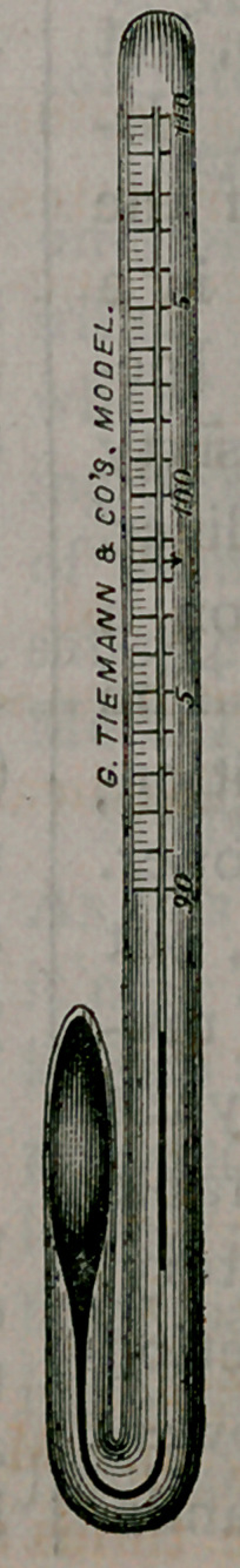# Abstracts and Gleanings

**Published:** 1877-04-20

**Authors:** 


### Abstracts and Gleanings.

VERATRUM VIRIDE IN PUERPERAL CONVULSIONS.—Much discussion was occasioned by a paper read by Dr. Fordyce, before Med. Association of Central New York, in which he claimed a peculiar adaptation of Veratrum Viride to the relief of convulsions resulting from uremia. He affirms "Albuminuria is the principal and the only pathological condition that for any length of time precedes convulsions; the same condition that produces the convulsions in albuminuria is, also, sometimes developed in pregnancy, and during the progress of labor by uterine pressure upon the bladder and ureters damming the urine upon the kidneys; the secretion of urine is suspended and urea retained in excess in the blood, &c.," and thus he accounts for puerperal convulsions. He then details a number of cases in practice as proof of the power of veratrum to control the convulsions. We extract from one of them the following quotation, which may serve to give an idea of his method of using the remedy in this formidable affection:

CASE I. The lady had nine convulsions and was comatose and insensible, breathing stertorous, etc., "advised Verat Viride, ten drops; repeat in thirty minutes in reduced doses until vomiting ensues, or pulse is reduced to sixty; convulsions controlled with second dose, and third dose reduced pulse to forty-eight; continue Verat. as soon as pulse increases above sixty; on the twenty-ninth, towards night, she became conscious, and by the use of Rochelle salts and Cream of Tartar and Acet. Pot. œdema disappeared; she became quite comfortable, and on the twenty-first of June, twenty-three days after attack was delivered of a still-born child in a stage of great decomposition, without convulsions or any unfavorable symptoms, so far as the mother was concerned."

SOLUTION OF SILVER IN THE TREATMENT OF ORCHITIS.—Our medical friend J. J. Knott, M.D., of Atlanta, (in Medical and Surgical Reporter), thus remarks: "From an extensive experience with the solution of nitrate of silver, I am satisfied that it is the most effectual remedy we possess for the speedy relief of this affection, forty-eight to sixty-four hours being sufficient to effect a cure in the majority of cases. My idea is that it operates in the same manner as strapping, viz,, by uniform pressure, thereby disgorging the blood vessels of the parts, and by this means relieving the inflamation. I commence the treatment by cleansing the scrotum with warm water and castile soap, and after drying the parts with a towel, apply the following solution with a camel's hair pencil:

R—Argenti nitrat (cryst.),......dr. ss
Aquæ destillatæ,.............oz. j
Fiat sol.

As soon as this application dries, apply the following;

R—Argenti nitrat (cryst),...grs. lxxx
Aquæ destillatæ.........oz. j
Sig.—Apply morning and night.

Should any excoriation of the scrotum follow this application, omit it at such points. My object in applying the thirty grain solution first is to protect the parts from the excoriating effects of the eighty-grain solution. Before I adopted this plan, I experienced some trouble on this account. Should the orchitis be complicated with secondary syphilis, the usual constitutional means should be employed in addition to the local treatment. J. J. KNOTT, M.D.

THE DISTINCTIONS BETWEEN THE ACTION OF CHLORAL AND OPIUM.—In his recently published work on Treatment, Dr. J. Milner Fothergill speaks as follows, in discriminating between the respective uses of opium and chloral:—"Chloral hydrate is a drug which stands second to opium only as an agent which depresses nervous action. There are differences, however, betwixt the actions of these two agents, which are far from unimportant. We have just seen that for the production of sleep two factors are necessary, viz: cerebral anæmia and a quiescent state of the cerebral cells. Opium acts more pronouncedly upon the cells than the circulation, whilst the effects of chloral are most markedly felt by the circulation, and to a less extent by the cells. Thus in old days a depressant, as tartar emetic, was combined with opium in conditions of sleeplessness due to vascular excitement. In such conditions chloral is the hypnotic par excellence. * * Where vascular pain and excitement co-exist, then chloral and opium should be combined. * * It also acts upon the cerebrum and the centres at the base of the brain, whilst it has a decided effect upon reflex irritability. From its double effect upon the nervous system directly and upon the circulation, chloral has been found very useful in the treatment of mania—much more useful than opium. Chloral too, is an excellent remedy in cases of cerebral irritability from overwork, giving calm, refreshing sleep."—[Medical and Surgical Reporter.

A NEW BLOODLESS OPERATION.—Wm. Powell, M.D., of Miss., (in Amer. Med. Bi-Weekly), thus describes a new bloodless operation:

A negro woman requested me a short time since to get a piece of needle ouof her arm, which she said had been accidentally lodged there about three years ago. I seated her near a table, applied a tourniquet buckled loosely about the middle of the arm, with the pad over the brachial artery; I then elevated the arm, with the hand higher than her head; an assistant pressed the pad, so as to stop the circulation in the artery; I then rubbed the hand and arm, forcing the blood from the limb through the veins to the body, then tightened the band of the tourniquet so as to prevent all circulation of blood in the limb beyond; I then laid the hand on the table, made an incision through the skin about an inch long and extracted a piece of needle half an inch in length. The tissues were entirely blanched; I dressed the wound before removing the tourniquet, and did not see a particle of blood, the knife was not stained. This case demonstrates very fully the fact that the bloodless operation can be performed on the extremities without any new contrivance, the ordinary tourniquet being fully sufficient.

NEW OBSERVATIONS CONCERNING THE THERAPEUTIC ACTION OF CARBOLIZED CAMPHOR.—Dr. Soulez publishes, in La France Medicale, of January 11, 1877, a series of cases in which he successfully employed the carbolized camphor. This antiseptic, of which he speaks so highly, is obtained by dissolving nine grammes of the crystals of carbolic acid in one gramme of alcohol; to two grammes of this solution is then added twelve grammes of powdered camphor—the syrup-like liquid which results is the carbolized camphor. For dressings the carbolized camphor is reduced (one part to nineteen of olive oil or oil of sweet almonds), and in the mixture are saturated square pieces of lint, which are then applied, one over the other, to the wound; these are covered by a sheet of thin india-rubber, and on this, again, dry lint is bound with a bandage. M. Soulez claims for this dressing three great advantages; suppression of pain after the operation, absence of febrile reaction, and, finally, rapid cicatrization.—Clinic.

PILLS OF SULPHATE OF QUINIA.—H. P. Reynolds, in the Amer. Journal of Pharmacy, recommends the following formula:

Quiniæ sulph. gr. 600; acid tartaric, gr. 100; glycerin, min. 75. Rub the quinia and acid together in a mortar to a fine powder till no appearance of crystals remains, add the glycerin—just seventy-five minims, no more, no less—and continue the trituration till the powder becomes adherent, when it should be beaten into proper form for handling and divided into the requisite number of pills. The mass is firm, solid, rolls well, does not set for some hours—is, in fact, a "beautiful mass," and the pills will be found quite small for their weight, very white if rolled in starch powder, and, however old or dry they may become, they remain perfectly and entirely soluble.—Pacific Medical Jour.

BAKING POWDER.—This is usually made by mixing three parts of dry sodium bicarbonate with three parts of farina, and intimately incorporating with it two parts of dry tartaric acid. The mixture must be put up and kept in air-tight cans or boxes, and must be well protected from moisture, as this would cause the acid to react upon the bicarbonate.—[New Remedies.

International Medical Congress—Section of Medicine.—Dr. J. J. Woodward, Surgeon U. S. A., Reporter, read a paper on "Typho-Malarial Fever; is it a Special Type of Fever?" After a general introduction, in which he referred to the duration of war as an element of mortality from sickness, and to the statistics of the Prussian army during their last great war, he discussed the subject of camp fevers in our army, especially when complicated with malarial influences. He sketched the history of typho-malarial fever, as reported by different surgeons, and argued that such hybrid combinations were not new in armies, illustrating this with historical facts He contended that typho-malarial is not a special type of fever; it is no new disease, but a hybrid. He referred at considerable length to those who had sustained his views, and to others who had opposed them.

Dr. Bartholow, of Cincinnati, replied to the charge of acrimony made by the reporter against him. He said he had simply objected to the erection, by Dr. Woodward, of a new type of disease.

Dr. N. S. Davis, of Chicago, said he saw little need of controversy; he was almost surprised that it was necessary to combat the idea of introducing a new disease. The phenomena of this mingling had been common to him. In Chicago, malarial disease was the common form; typhus, when it did exist, was imported. In the progress of filling up the city, the malarious influence had gradually disappeared, and for ten years typhoid was gradually on the increase, owing to the peculiar water arrangements. After a time typhus had predominated. To him it was a surprise that any criticism should be made concerning the two diseases being present at the same time. The sooner we recognize this capability for mingling, among diseases, the sooner will we clearly recognize and understand the mingling in this case.

Dr. Scott, of Ohio, said he had always lived in a malarious country, and recognized this complexity and union of diseases. Early, it was true, this mingling was not so pronounced, but as civilization advanced, it rapidly grew to be so. He thought these discussions were of use, because we do not understand the treatment of the affection. Quinine will not check it, although it can be used to check the malarious condition. He had seen scurvy, while in Nashville, in 1864, in connection with this disease, and he therefore agreed with Dr. Woodward.

Dr. Pepper said he feared the employment of specific terms in such cases, especially as it concerns young men, for it gives rise on their part to a lack of knowledge of the real danger present.

Dr. Reed, of Camden, objected to a term that signifies two specific affections.

On motion, the paper was referred to the Section for publication.

[We had a case of the above character, recently, wherein there was decided intermissions and sweats, with red, dry tongue. Quinine made no favorable impression after ten days' use, but rather irritated the stomach. The case yielded, after four days use, to Fluid Ex. Eucalyptus, ten drops every three hours. Suspended during the night, and the free use of lemonade allowed, the patient having a thirst for acids. W.]

Treatment of Coxalgia.—Prof. Lewis A. Sayre, Reporter, then read a paper on this subject, which elicited considerable discussion. The following were the conclusions deduced from it:

1st. Morbus coxarius is a disease peculiar to early childhood, or the age of reckless indifference.

2. It is almost always of traumatic origin, and not necessarily connected with vitiated constitution.

3d. Rest and freedom from pressure of the parts involved, while at the same time the rest of the body is allowed free exercise in the open air, and a nutritious diet, is the best treatment yet devised.

4th. If this plan of treatment is adopted in the early stages, the majority of cases will recover, with nearly if not perfect motion, and without deformity.

5th. In the advanced second stage, when absorption cannot be produced, then it is better to puncture or aspirate the joint and remove its contents, than to leave it to rupture by ulceration.

6th. In the third stage, where the treatment here recommended has been properly applied without satisfactory improvement, but progressive caries continues, then exsection of the diseased bones is not only justifiable, but absolutely necessary.

7th. The operation of exsection of the hip is easily performed, and attended with no danger.

8th. After exsection of the hip-joint in cases of caries, the recovery is much more rapid and certain, and infinitely more perfect as to form, motion, and the usefulness of the joint and limb, than when left to the slow process of nature's exfoliation.—Med. Record.

ANÆSTHESIA.—Dr. Addinell Hewson, of Philadelphia, read a paper on "Anæsthesia, as produced by Nitrous Oxide and Rapid Breathing."

He stated the method had been suggested to him by W. G. Bonwell, a dentist of Philadelphia, and he had tried it in a number of cases, in public and pri vate practice, with varying but satisfactory results. The method pursued was to cause the patient to breathe from forty to fifty times in the minute, the effect of which in from three to five minutes was a tingling of the surface, with flush face. Consciousness remained unimpaired, and the patient would perform any act desired, but was rendered totally devoid of sensibility. This method would be advantageous for short minor operations, especially those about the nose, throat, etc. The process occupied a longer time in young people and in cold weather. He explained the theory of its action by the retention in the blood of carbonic acid.—Med. Rec.

INUNCTIONS IN SYPHILIS.—Inunctions are admitted by Fournier to be useful in the following cases:

1. In case of serious lesions, which it is necessary to act upon surely and rapidly.

2. In cases which are rebellious to other methods.

3. In cases in which mercury is not tolerated by the stomach.

There can be no question but that inunction is the method *par excellence* in iritis. Cases are not infrequently met where, the internal use of mercury having proved of no avail, the employment of inunctions is followed by a rapid and satisfactory result.—DR. HARLINGEN in Med. Times.

ANTIDOTE FOR CARBOLIC ACID.—As this acid is now so extensively used, it may be of some importance to make known the antidotes which have been proposed. M. Ferrand advises the following: white sugar, fifteen parts; water, forty parts; quicklime, five parts, forming a sacharate of lime.—Oregon Med. Journal.

AGENTS AFFECTING THE SECRETON OF THE BILE.—Professor Rutherford and M. Vignall have continued their observations on cholagogue drugs. They employed euonymin, sanguinarin, iridin, leptandrin, ipecacuanha, colocynth, and jalap. The animals used for experiment were invariably dogs, and each experiment lasted an entire day. The various substances were always injected directly into the duodenum. 1. In regard to euonymin, it was found that five grains mixed with boiling water powerfully stimulated the liver. It is an active purgative in the human subject. 2 In regard to sanguinarin, three grains, and one grain in two different experiments, mingled with a little bile, powerfully stimulated the liver, but rendered the bile more watery, though more biliary matter was secreted in a given time. The secretion of the intestinal glands was slightly increased. 3. In regard to iridin, five grains mixed with a little bile and water very powerfully stimulated the liver. It is not so powerful as large doses (four grains) of podophyllum, but it is more powerful than euonymin. Irdin is also a decided stimulant of the intestinal glands. 4. Leptandrin is a stimulant, but only a feble one. 5. Ipecacuanha, when given in doses of sixty grains, powerfully stimulated the liver. Even three grains had an effect on a dog weighing seventeen pounds. The bile secreted was of normal composition, as regards the biliary matter proper. No purgative effect was produced, but there was an increased secretion of mucus in the small intestine. 6. Colocynth is a hepatic stimulant of considerable power. It renders the bile more watery, but nevertheless increases the secretion of biliary matter. It is also a powerful stimulant of the intestinal glands. 7. Lastly, the results of the experiments with jalap showed that the drug is a hepatic stimulant of considerable power. It renders the bile more watery, but at the same time increase the secretion of biliary matter. Its effect on the liver is, however, far less notable than its effects on the intestinal glands.—The Journal of Anatomy and Physiology.

THE BLUE LIGHT CURE.—It has been remarked that this is an age of progress and invention. The latest excitement in the way of remedial agents is what is termed as above. It consists in placing the patient in a position where the sun's rays, striking through blue glass, will fall upon the afflicted part. The most remarkably beneficial results are attributed to this novel treatment, and the numerous cures effected by it are beginning to attract the attention of the medical profession. Several hospitals are being fitted with blue glass window panes, which are arranged alternate with the white ones. It is not an unusual thing to see huge plates of blue glass displayed for sale in stores whose trade is other than in window glass. We presume that druggists will be obliged to go into this new "remedy," and get a stock of blue glass on hand, to supply the demand.—Drug. Advertiser.

HOW TO MAKE LEECHES BITE.--A Mr. James P. Myles, of Parsonstown (Dub. Med. Press and Circ.), has always made leeches bite by placing them in an apple properly hollowed out, and applying them over the part.

SULPHO CYANIDE OF POTASSUM, has the property of rendering old and faded letters legible. First make a solution in water and dip the letter into it, and while damp expose to the fumes of hot hydro chloric acid.

THERMOMETRY IN DISEASES.—We fear that too many practitioners are neglecting the use of the thermometer as a means of diagnosis and prognosis. The axilla is the best part for ascertaining the temperature of the body. The rectum is also a good part for determining the temperature. An increase of 1° of Fahr. corresponds with an increase in the pulse of ten beats per minute. The range of temperature in health is from 97-92° to 99°. In fevers the thermometer may mark 106° 108° in the armpit. When it stands 98.50° it shows derangement of health When it reaches 101°–105° the fever is severe: if above 105° the patient's condition is dangerous; with 108°–109° death will ensue in a short time.

In typhoid fever a temperature which does not exceed in the evening 103.5° indicates a mild course of fever. 105° in the evening and 104° in the forenoon indicates a protracted and dangerous case. In pneumonia 104° marks a severe attack—a like temperature in rheumatism is alarming. A falling temperature from evening to morning is favorable but the reverse is unfavorable.

EAU DE COLOGNE AS AN ANÆSTHETIC·—At a recent meeting of the Nice Society of Medicine, Dr. Hugues presented some observations upon the anæsthetic influence of Eau de Cologne, which he had recently noticed. In one instance, that of a young lady afflicted with tubercular consumption, and with whom injections of morphine and the use of chloral had failed to produce the desired repose, a friend suggested a trial of eau de cologne, which she had already used with success in similar circumstances on some twenty different occasions. An immediate experiment was made, by placing a handkerchief well moistened with cologne under the nostrils of the invalid, who, in the space of seven minutes, sank into a profound slumber. The same experiment was repeated in other cases, with excellent results.—Med. and Surg. Reporter,

THE INDIANAPOLIS SUN has taken high rank among the political newspapers of the country, and is edited with marked ability. It is fearless and candid in the discussion of leading questions of reform, especially in currency and finance. For terms see advertisement elsewhere.

TYPOGRAPHICAL ERRORS.—We have carefully endeavored to avoid these, but in the hurry and confusion which exists in our large printing establishments such things must be expected occasionally. Several occurred in our last issue which we trust to the good sense of our readers to correct.

TIEMANN & CO'S NEW THERMOMETER.—GEO. TIEMANN & CO., 67 Chatham St., New York, inventors and manufacturers of the thermometer represented above, claim for it:

1. The registering portion of index cannot be united with the main column of mercury in the bulb except by design. The device of the "bend" fulfils the object of guarding against accidental loss of Index.

2. The scale is graduated in ¼°, and is identical in every respect with that of a *four*-inch thermometer, whereas the bent thermomoter is less than 3¼ inches in length.

3. The portion of the thermometer intervening between the bulb and the commencement of the scale lies in juxtaposition to the bulb, and the ascending mercury is therefore subjected to the warmth of the parts as well as the bulb, and is not exposed to external temperature.

4. It will not roll. The advantage of this latter feature will be readily recognized and appreciated.

The thermometers are safely carried in a neat morocco case (lancet case style), lined with velvet, and can be conveniently placed in the vest pocket, being but 3⅜ inches in length, and but ¾ inch wide; price $3.50.

ACONITE IN HEADACHE.

Aconite is adapted to the treatment of congestive headaches attended with cold extremities. It acts by dilating the peripheral bloodvessels and thus relieves the cerebrum.

CHLORAL HYDRATE IN TETANUS.

Cases of Tetanus treated successfully with the above agent are reported in the Journals. As recovery from this affection is extremly rare it is well to note the above fact. Very large doses are required. In one case the patient having suffered three days without any relief, and there being scarcely a ray of hope, ten grammes (150 grs.) of the remedy were thrown into the rectum. The patient obtained some sleep, and next day the dose was repeated—again followed by improvement. It was continued from day to day, both by mouth and rectum for ten or twelve days, until the patient was convalescent.

## Figures and Tables

**Figure f1:**